# Implementation of a Healthcare Implicit Bias Training across Three Oncology Care Settings

**DOI:** 10.1007/s13187-025-02779-9

**Published:** 2025-11-23

**Authors:** Dana Rosenberg, Teletia Taylor, Hannah Arem, Kimberly Davis, Carla Williams, Julie E. Bauman, Christopher J. King, Mandi L. Pratt-Chapman

**Affiliations:** 1 GW Cancer Center, The George Washington University School of Medicine and Health Sciences, Washington, DC United States; 2 Howard University Cancer Center, Washington, DC United States; 3 Healthcare Delivery Research, MedStar Health Research Institute, Washington, DC United States; 4 Georgetown Lombardi Comprehensive Cancer Center, Washington, DC United States; 5 Department of Medicine, The George Washington University School of Medicine and Health Sciences, Washington, DC United States

**Keywords:** Implicit bias, Oncology, Implementation strategies

## Abstract

The purpose of this study was to describe evaluation outcomes, including reach, satisfaction with training, and strategies to increase uptake of implicit bias training across three cancer centers in Washington, DC. From October 25, 2022, to March 29, 2023, oncology clinicians and staff were asked to complete an implicit bias training and post-training survey. Strategies used to increase training reach included identifying and preparing champions, identifying facilitators and barriers, promoting adaptation, and mandating completion. We quantitatively assessed satisfaction with training and self-reported learning outcomes and qualitatively explored impact of the training and how implementation strategies affected uptake. We reviewed open-text responses for feedback and insights from learners. At the suggestion of our champions, we pivoted from a five-hour training to a one-hour training to adapt to clinical team availability. The most successful strategies for increasing training uptake included reducing training length and personalized follow-up from institutional champions. One hundred eight of the 163 (66%) invited learners completed the training. Over 94% of learners agreed or strongly agreed that they met the training’s three learning objectives: describing implicit bias, providing examples of implicit bias in healthcare settings, and reflecting on one’s own biases. Learners’ open-ended responses described how training reaffirmed viewpoints for some learners while others became more aware of their biases. Implementing an implicit bias training across multiple care settings was successful in reaching our goal of 100 learners; however, reach varied by setting due to competing priorities and less champion influence at one institution.

## Introduction

Racial and ethnic disparities in cancer health outcomes are well known in the U.S [1]. The cause of disparities is complex and multi-level, including entrenched bias that manifests in differential social determinants of health for various racial and ethnic groups and racially discordant clinicians (non-Black) and patients (Black) interactions [2]. Perceptions of bias in oncology practice vary widely, with younger generations more often noticing racial anxiety and bias than older generations [3]. Past research has suggested that implicit bias during clinical interactions between racially discordant patients and clinicians has important effects on communication including shorter clinical interactions, less patient retention of information, and lower patient confidence in recommended treatments [4].

In 2020, the American Medical Association advocated for medical schools to implement implicit bias training [5]. There has also been a push by legislators to mandate implicit bias training for health care providers [6]. In fact, eight states require implicit bias training to be included in medical education requirements [7]. In Hagiwara et al.’s (2024) systematic review of 77 studies investigating implicit bias training in the health care setting, it was reported that despite the increasing number of health care organizations incorporating implicit bias training, there are no clear guidelines for the development and implementation of training [6]. In a scoping review of post-graduate physician implicit bias training, Gleicher et al. found that implementation facilitators and barriers such as institutional support of implicit bias training and competing demands for medical staff time were important barriers when considering implicit bias training uptake among post-graduate physicians [8].

In response to these calls for action, three cancer centers in Washington, DC collaborated to test a multi-level intervention to improve social care connections and reduce bias in oncology practices through the Building Equal Access Together in Cancer (BEAT-C) study. BEAT-C is a multi-level study in which we first conducted interviews with health care professionals and patients to inform workflow for Health Related Social Needs (HRSN) screening and referral for cancer survivors in Washington, DC; and we also developed Diversity, Equity, Inclusion, Justice (DEIJ) Task Forces to improve patient-centered systems of care, which we have previously described [9–11]. We are currently investigating the impact of community health worker (CHW) support on cancer survivor health and wellbeing as measured through quality of life and social connection.

A secondary aim of BEAT-C was implementing and evaluating an implicit bias training for staff and clinicians across the three cancer centers, which is the focus of the present analysis. We utilized an existing, publicly available health care professional training Implicit Bias II: A practical guide for healthcare settings [12]. We summarized learner confidence in learning objectives, learner satisfaction, and open-ended feedback post-training for learners specifically targeted for training between October 25, 2022 and March 29, 2023. Here, we describe implementation strategies to increase training reach and satisfaction outcomes for the implicit bias training of clinicians and staff across three cancer settings.

## Methods

### Ethical Review 

The Georgetown-Howard Universities Center for Clinical and Translational Sciences institutional review board approved the study (Study 4397).

### Study Population 

Clinicians and staff working directly with patients within oncology clinics where BEAT-C patients were treated were invited to take the training. The study team created a targeted roster of 163 oncology clinicians and staff across three sites who directly worked with cancer patients day to day.

### Training Description

The research team originally selected a five-hour, Together-Equitable-Accessible-Meaningful (TEAM) training, developed by the study MPI [13]. Subsequently, the research team also identified an existing, virtual, self-paced one-hour implicit bias training developed by the D.C. Department of Health titled Implicit Bias II: A Guide for Healthcare Settings [12]. The training used a series of five case-based scenarios to discuss implicit bias concepts and examples. Learning objectives included: describe how implicit bias can negatively impact health outcomes, identify examples of implicit bias in healthcare settings and their root causes, and reflect on one’s own biases and develop strategies to overcome biased decision-making [12]. In our results, we only included those who completed this specific training.

### Delivery Strategies

 Cancer center directors at the three sites were asked for guidance on disseminating and implementing the training. Multiple Expert Recommendations for Implementing Change (ERIC) strategies were used to increase uptake of implicit bias training among clinicians and staff [14]. These strategies included: (1) identifying and preparing champions, (2) identifying facilitators and barriers, (3) promoting adaptability, and (4) mandating completion of the training (where possible).

### Data Collection

 Post-test learner data on satisfaction with training and learning outcomes were collected online through the George Washington University School of Medicine and Health Sciences Learning Management System (LMS) (see Table 3). In order to access the training, each learner had to create an account that included their name, basic demographic information, and email address. All data on learning outcomes were based on data directly entered by the learner into the LMS. Tracking of implementation strategies was done collectively via a shared LISTS tracking log accessible on Medstar Box [15].

### Data Preparation

Data were downloaded from the LMS to Excel inclusive of content from the launch of the training (June 6, 2020) through the date of download (November 12, 2024). A subset of data (*n* = 163) was generated by restricting the data set to only learners from the three original institutions participating in the BEAT-C intervention who completed the training during the implementation push from October 25, 2022 to March 29, 2023. Results from *n* = 330 individuals across the three institutions are not included here because they were not part of our focused implementation efforts directed to frontline oncology staff and clinicians. For example, individuals who comprised these additional learners included medical students as well as clinicians and staff in non-oncology departments. The post-training survey included Likert scale questions about satisfaction with learning objectives, style, format, level of training, and applicability to practice. The Likert scale items were converted from descriptive labels to numbers as follows: Strongly Agree = 5, Agree = 4, Neutral = 3, Disagree = 2, Strongly Disagree = 1. Data are displayed in Table 3.

### Data Analysis

Descriptive statistics for learners were aggregated in Table 1. We categorized learners based on their role and whether they were clinicians or staff. Clinicians included physicians, dieticians, a genetic counselor, nurses, nurse practitioners, patient navigators, physician assistants, and social workers. Staff roles included administrators, researchers, and staff such as call agents and scheduling associates.

Satisfaction with training was compiled using descriptive data (Table 3). Short, open-ended responses to questions about learner experiences with the training were were coded for emergent themes. Codes were populated in Excel and NVIVO 15 software with exemplar quotations. The MPI verified the thematic coding and analysis for concurrence. Implementation strategies used to optimize reach were also described.

## Results

Overall, *n* = 108 learners across three BEAT-C cancer care organizations completed the intended training. There were two trainings offered in the online LMS with similar course descriptions and titles, Implicit Bias II: A Practical Guide for Healthcare Settings and Implicit Bias. Due to confusion in having two trainings with the name “Implicit Bias” in the title, an additional nine individuals across the three institutions took the incorrect (Implicit Bias) training during the implementation push, so their results are not included in this analysis. Table 1 shows characteristics from the sample of learners who completed the intended Implicit Bias II: A Practical Guide for Healthcare Settings training during the focused period of our intervention, broken down by clinicians and staff roles, as well as occupation.

**Table 1:** Learner occupation characteristics for [name of training] (*n* = 108)

Clinician/Staff Designation	Occupation	Percentage who Completed the Training *N* (%)
Clinician	Attending Physician	51 (47)
Clinician	Dietician	2 (2)
Clinician	Genetic Counselor	1 (1)
Clinician	Nurse	18 (17)
Clinician	Nurse Practitioner	4 (4)
Clinician	Patient Navigator	7 (6)
Clinician	Physician Assistant	3 (3)
Clinician	Social Worker	4 (4)
Staff	Administrator	1 (1)
Staff	Researcher	7 (6)
Staff	Staff	10 (9)

Of those asked to complete the training, *n* = 108/163 completed the training, representing a 66% percent overall completion rate across all institutions. Participation varied by site with Institution 1 having the highest completion rate of 89% (*n* = 50/56), Institution 2 at 68% (*n* = 47/69), and Institution 3 with the lowest completion rate of 29% (*n* = 11/38), see Table 2.

**Table 2:** Participation by site

Site	Invited	Completed of those invited and eligible	Percent completed of invited learners
Institution 1	56	50	89%
Institution 2	69	47	68%
Institution 3	38	11	29%

## Delivery Strategies

With regards to ERIC strategy 1, identifying and preparing champions, Site PIs met with cancer center directors at each institution and championed completion of the training at their institution. In a separate but synergistic initiative, the Site PIs convened 4–5 cancer center faculty and staff from diverse backgrounds (e.g., physicians, nurse managers, community health workers, cancer committee members and cancer registry coordinators, and research associates) to comprise a Diversity, Equity, Inclusion, Justice (DEIJ) task force at each of their respective institutions [9]. The three institutional task forces were designed to be flexible and responsive to volunteer faculty committed to advancing DEIJ in the context of oncology; thus, the composition of the group and goals of each group were unique. Two of the three institutions prioritized implicit bias training as a top priority, and thus members from these two institutional task forces served as champions to encourage peers to complete the implicit bias training at their respective institutions. Other activities of the DEIJ task forces are described elsewhere [9].

For strategy 2, identifying facilitators and barriers to implicit bias training, we met with institutional champions as well as those who were asked to take the training. A major facilitator of training completion was buy-in from cancer center leadership. For example, at Institution 1, there was a mandate from the cancer center director for training completion. At Institution 2, there was “strong encouragement” to complete training from the cancer center director, dean of the medical school, and the site PI. Increased completion was facilitated by following up with learners who had not completed the training and engaging supervisors if several attempts at requesting completion failed to yield results (Institution 1). Another facilitator of training completion was use of relevant content through relatable scenarios that enabled learners to apply knowledge to immediate clinical care interactions. A barrier to training completion was competing demands. For Institution 3, the clinical staff already felt overwhelmed from the number of required trainings; and in the process of disseminating our training, staff were notified that their institution was planning to make a different implicit bias training mandatory. All invited learners for institution 3 were invited to take the Implicit Bias Training: A practical guide for healthcare settings before their institutional leadership indicated a different, private training was required on the same topic. We did not press employees of institution 3 to take the training given this internal requirement. This made buy-in more challenging and resulted in a lower percentage of clinicians and staff completing the BEAT-C training at this institution (29%).

For strategy 3, promoting adaptability, after meeting with leadership, the original intervention was changed from a proposed five-hour training on intersectional health equity to a one-hour, case-based training. Additionally, the nine individuals who completed a different implicit bias training within the LMS were not asked to take another training given that both trainings had similar learning outcomes and objectives.

## Satisfaction with Training

Satisfaction with training was high among learners with mean scores in the agree/strongly agree range for learning objectives and training presentation outcomes (see Table 3). Further, 94% (*n* = 102/108) participants indicated that the training: (1) was presented at their level of comprehension and was communicated in a clear and effective fashion, and (2) the style and format for the presentation of each module was well-suited for their on-line learning needs.

**Table 3:** Learning outcomes and satisfaction with Implicit Bias II: A Practical Guide for Healthcare Settings (intended intervention) (*n* = 108)

Item	M (SD)
Describe how implicit bias can negatively impact health outcomes.	4.58 (0.66)
Identify examples of implicit bias in healthcare settings and their root causes	4.52 (0.65)
[The training helped you] reflect on [your] own biases and develop strategies to overcome biased decision-making.	4.50 (0.68)
The major concepts of this module were presented at your level of comprehension and communicated in a clear and effective fashion.	4.49 (0.70)
The key points were made relevant to your practice through the use of descriptive scenarios that enables you to apply the knowledge in decision-making.	4.45 (0.77)
The information presented in this module conveys knowledge that is important to you and will enable you to improve aspects of your practice.	4.44 (0.77)
The style and format for the presentation of this module was well-suited for your online learning needs.	4.51 (0.70)

Likert scale: Strongly Agree (5) to Strongly Disagree (1)

## Intention To Change Practice

The post-training survey asked learners what changes they would make to their practice as a result of the training. Many respondents expressed they would be more aware and mindful of their biases when interacting with patients. Several learners emphasized individual responsibility in changing their practice. For example, one clinician stated, “I will attempt to be more mindful and aware of my own biases.” Others highlighted the impact on patient care including a staff member who said, “I will be more mindful in how I ask questions about preferred language and insurance status.” A smaller number of learners indicated they would make changes to their clinic intake process, standardize patient processes, and/or encourage colleagues to take the training. Of note, these questions were asked immediately after the training and there was no way to assess follow-up to these actions post training.

When asked how the learning activity changed the learner’s view on implicit bias, many respondents indicated the training helped increase self-awareness and understanding of implicit bias and related concepts. In terms of awareness, some respondents expressed they would be more self-aware as a result of the training, similar to how learners reported they would change their practice. One clinician stated the training “alerted me to be more in tune with my own biases.” Several other respondents recognized the universal nature of implicit bias, including a clinician who described “implicit bias is real in each of us” and a staff member who stated, “everyone has implicit bias.”. Additionally, several people mentioned the training reinforced prior learning about implicit bias. One clinician learner said, “We need to educate and re-educate ourselves constantly.”

Some respondents indicated they had taken previous implicit bias trainings and were familiar with implicit bias concepts. Other respondents felt the training “did not change anything” or had no response regarding if the training changed their viewpoint or their practice. Still, some of the learners did appreciate the refresher including a clinician that noted the training “provided a refresher on how to treat patients as individuals and with respect.”

When asked how patient outcomes might be improved because of the training, most learners indicated that they anticipated improved patient satisfaction as a result of enhanced communication, relationships, and trust with the medical environment and team. One clinician shared they anticipated that “patient’s [will] feel heard, seen, validated, and share with their healthcare professional in a way that optimizes the patient’s health.” Learners indicated personal intentions to be more humble, empathic, and sensitive and specifically called out race, cultures, microaggressions, and gender identity as areas of focus. One learner said, “It will help make patients more comfortable and valued in the health care system.”

Requests for additional training included content on additional concepts related to implicit bias (Implicit Association Test, racial profiling, microaggressions, health disparities), LGBTQ-affirming care, clinical trials, and strategies for caring for patients with mental disabilities. When asked about learning preferences, 81% of respondents preferred online continuing medical education (CME).

## Discussion

We found that a short, applied training was largely acceptable to oncology clinicians and staff and that uptake of training increased when an institutional leader personalized requests for completion. Our study contributes to the literature by reporting on how implementation strategies, including leadership buy-in and active promotion, adaptability in training format and dosage to fit learner needs, and mandate of completion from leadership optimized training completion for our targeted group.

Recent studies have shown a relative paucity in implicit bias training among healthcare professionals, despite known bias that differentially influences health care outcomes [16, 17]. Our study showed the feasibility of widespread implicit bias training completion by oncology clinicians and staff when directly requested to complete training from institutional leadership (*n* = 108/163). Institutions 1 and 2 attained a high training completion rate (82% for Institution 1, 68% for Institution 2). However, Institution 3 was only able to attain a 29% completion rate for the reasons stated above. These results align with positive feasibility reported by another recent study of implicit bias training in oncology led by the American Society of Clinical Oncology [18]. Additionally, this builds on a recent scoping review that identified lack of institutional support and competing time demands for health professionals as barriers to implicit bias training implementation [8]. For Institution 3, the fact that completion was so low prior to a separate mandatory requirement for a different training suggests that competing priorities and training burnout may lead to lower completion rates unless trainings are required and championed by institutions.

In a rapid review, Fricke et al. (2024) evaluated the effectiveness of healthcare worker implicit bias training and reported that there was no information on barriers and facilitators of implicit bias training, nor was there information about personnel, time, or resources required to implement training [19]. The current report begins to fill this gap by providing information on facilitators and barriers we identified when implementing implicit bias training. In terms of resources, we adopted an existing training developed by one of the co-investigators of the study, thus minimizing new resources needed to achieve our intended goals. The personnel time required to meet with cancer center leadership, identify intended learners, invite learners, monitor completion of training, and send learners reminders to complete training was able to be integrated into existing staff roles.

### Limitations

Originally, we intended to evaluate learning with a pre/post intervention validated scale and learning questionnaire. By embracing adaptability needed to accomplish our goals for reach, we were limited to evaluating the data already pre-programmed into the LMS. We were unable to add to these evaluation questions, thus limiting our results to reach and post-test findings only. Additionally, we were not able to follow up with trainees about their attitudes and behaviors after the post-test was administered.

### Future Directions 

Relatively little is published on the impact of implicit bias training in cancer centers. Future studies should explore pre-/post- learning change, association of total training time with learner outcomes, and actual change to practice.

## Conclusion

While online continuing education is only one way to raise awareness and encourage practice change, our study showed the feasibility of using online CME reaching a large number of individuals working across multiple cancer centers.

## Data Availability

De-identified data will be made available to academic researchers wishing to use data for non-commercial purposes upon the request to the corresponding author.

## Declarations

### Permission to Reproduce Material

No copyrighted material, surveys, instruments, or tools were used in the research described in this article.
